# The Use of Spectroscopy Handheld Tools in Brain Tumor Surgery: Current Evidence and Techniques

**DOI:** 10.3389/fsurg.2019.00030

**Published:** 2019-05-29

**Authors:** Nikita Lakomkin, Constantinos G. Hadjipanayis

**Affiliations:** ^1^Department of Neurosurgery, Mount Sinai Health System, Icahn School of Medicine at Mount Sinai, New York, NY, United States; ^2^Department of Neurosurgery, Mount Sinai Health System, Icahn School of Medicine, New York, NY, United States

**Keywords:** handheld technologies, gliomas, fluorescence-guided surgery, brain tumors, 5-ALA = 5-aminolevulinic acid, Raman spectroscopy

## Abstract

The fundamental principle in the operative treatment of brain tumors involves achieving maximal safe resection in order to improve postoperative outcomes. At present, challenges in visualizing microscopic disease and residual tumor remain an impediment to complete tumor removal. Spectroscopic tools have the theoretical advantage of accurate tissue identification, coupled with the potential for manual intraoperative adjustments to improve visualization of remaining tumor tissue that would otherwise be difficult to detect. The current evidence and techniques for handheld spectroscopic tools in surgical neuro-oncology are explored here.

## Introduction

The fundamental goal in the surgical treatment of brain tumors is achieving the greatest possible extent of resection while minimizing injury to surrounding pathways present in the adjacent brain. Indeed, numerous studies in the literature have demonstrated that the proportion of tumor removed is significantly associated with key postoperative outcome metrics, including overall survival (OS) and progression-free survival (PFS) (1–3). Despite this, however, complete tumor resection remains challenging due to the presence of microscopic disease and infiltrative tumor tissue that is difficult to visually differentiate from the surrounding brain. As such, numerous tools have been developed, for both pre- and intra-operative use [e.g., neuronavigation and intraoperative MRI (iMRI)], in order to assist in the identification and removal of neoplastic tissue (4). More sophisticated MRI techniques, such as whole brain magnetic resonance spectroscopic imaging (MRSI), has allowed for more precise visualization of tumor than would otherwise be possible with standard MRI (5). Multiple studies have highlighted the fact that microscopic involvement of high-grade gliomas (HGGs) extends well-beyond the contrast-enhancing lesion visible on T2-weighted MRI (5, 6). Other techniques, such as neurosurgical virtual reality and simulation have facilitated preoperative visualization and planning (7). Although these instruments have demonstrated utility, intraoperative retraction and tumor resection often result in brain shift, making it challenging to assess the extent of resection in real time. Also, non-specific enhancement after iMRI may be confused with residual tumor. Fluorescence-guided surgery using 5-aminolevulinic acid (5-ALA) has been a crucial addition to the neurosurgeon's toolkit, allowing for direct fluorescence visualization of HGGs using wide-field surgical microscopy (8). However, the infiltrative margin of HGGs and World Health Organization (WHO) grade II low grade gliomas (LGGs) have remained challenging to visualize, since sufficient levels of fluorescence often cannot be detected using traditionally employed visualization technologies (9). Handheld tools were developed as an adjunct to intraoperative resection, under the premise that real-time use and the ability to adjust the angle of the instrument could facilitate detection of remaining tumor at the time of surgery. Among the most studied techniques has been the use of handheld Raman spectroscopy in the identification of cancer cells during surgery. Other devices aimed at improving visualization in fluorescence guidance surgery have recently been developed. The purpose of this review is to examine the technique and evidence for the utility of handheld spectroscopy tools in neuro-oncologic surgery.

## Technique

### Raman Spectroscopic Probes

Handheld Raman spectroscopic probes harness the traditionally employed Raman spectroscopic technique described decades ago (10). Monochromatic light from a laser is used to shine on an object of interest, resulting in a change in vibrational energy states, which can be detected as electromagnetic radiation that is filtered through a monochromator (11). This results in a molecular fingerprint that can be subsequently harnessed to differentiate various tissue types (12). Neoplastic cells, for instance, have a molecular composition that is distinct from normal brain parenchyma and thus allows for their identification during surgery (13, 14). Glioblastoma (GBM) has been shown to exhibit a decreased lipid band and an increased protein band, while cholesterol bands were found to be absent in metastatic brain lesions (15). One specific advantage of this technique is that water molecules do not interfere with the Raman scattering, thereby increasing its utility in surgical applications. In the operating room, the laser/collection cones, lens, and filter can be combined into a hand-held probe that is then connected to a camera and spectrometer device. This probe can be used intraoperatively during resection in order to both identify tumor cells outside of contrast-enhancing areas on anatomic MRI as well as residual tumor following debulking and resection (16). Unlike the MRI neuronavigation, virtual reality, and other imaging techniques, the handheld probe allows the surgeon to make intraoperative adjustments in terms of positioning and angles following changes in the relative location of anatomic structures during surgery.

### 5-ALA Fluorescence Visualization

**Figure 1:**
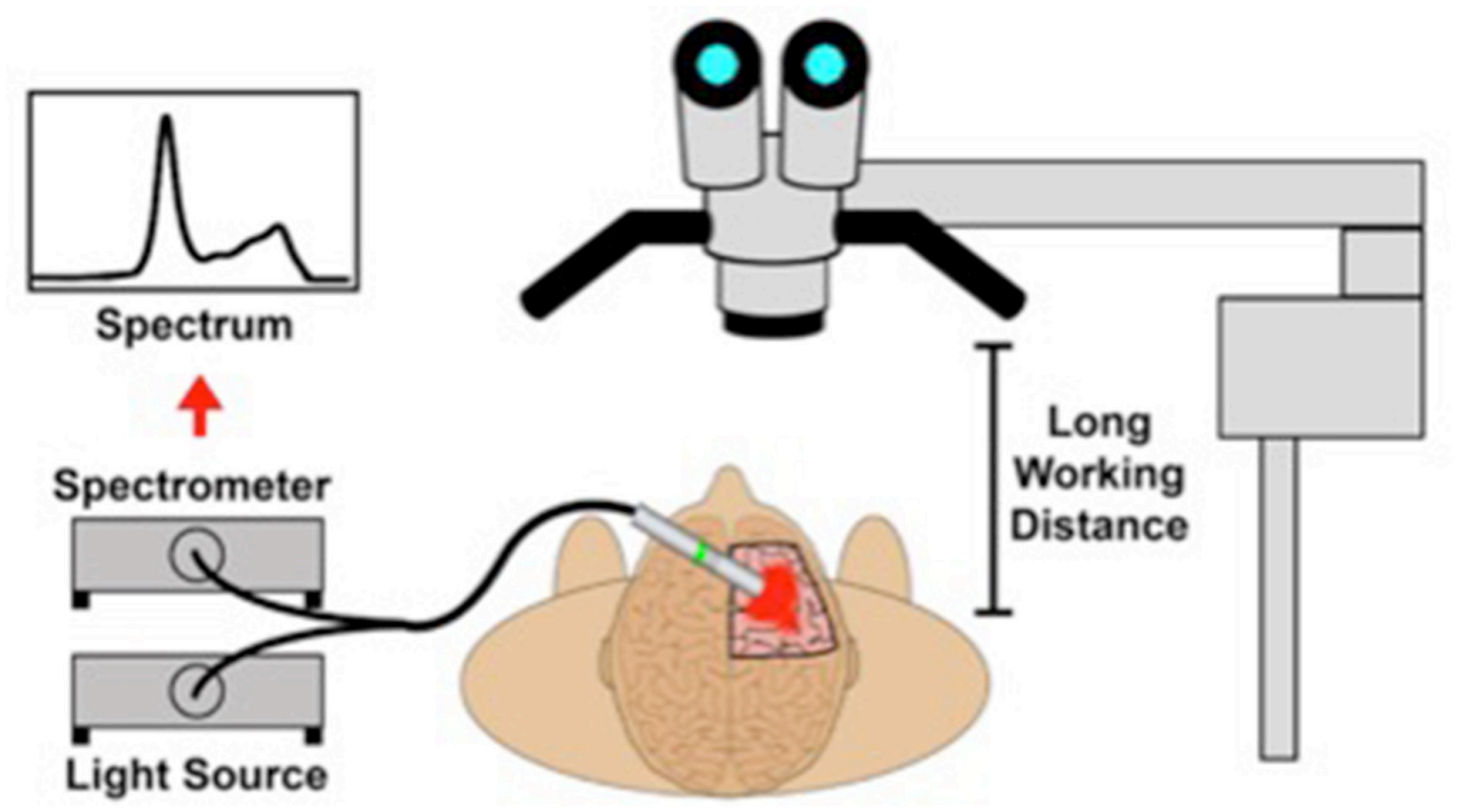
A schematic depicting the intraoperative use of a handheld spectroscopic probe in order to quantify relative protoporphyrin IX levels in various tissues [With permission from Kairdolf et al. (25)].

5-ALA is an oral pro-drug and the preoperative administration of this agent results in the accumulation of the fluorescent metabolite protoporphyrin IX in neoplastic lesions, which can be visualized during surgery (17, 18). This is accomplished via excitation of protoporphyrin IX-rich tissue with blue 440 nanometer wavelength light, resulting in the emission of a violet-colored signal (19). Improved visualization of tumors consequently allows for a greater extent of resection than would otherwise be possible under white light. Numerous randomized, controlled trials have demonstrated a benefit to patient survival using this agent, resulting in it gaining recent FDA approval for the treatment of suspected HGGs in the United States (20–22). Despite this, however, the evidence for its utility in the treatment of LGGs is less robust, due to the difficulty in achieving adequate fluorescent signal using wide-field microscopic illumination from a long working distance (9, 23). In addition, visualization of the infiltrative tumor margin becomes more difficult with fluorescence due to the lower number of tumor cells residing away from the tumor bulk. Handheld probes offer distinct advantages in visualizing these tumors, due to both the ability to place the instrument in close proximity to the tissue, as well as potentially generate a precise, quantitative measurement of protoporphyrin IX concentrations (24, 25). These techniques involve utilizing a handheld probe coupled to spectrometer that can be manipulated within the intraoperative field (Figure 1) (25, 26).

## Evidence

### Raman Spectroscopy

#### Basic Science

An array of *ex-vivo* and animal model studies have demonstrated the value of handheld Raman spectroscopy in differentiating tumor from normal brain tissue. Ji et al. examined the ability of Raman microscopy to discriminate tumor samples from 22 biopsy specimens, and found that Raman spectroscopy detected tumor infiltration in near-perfect agreement with hematoxylin and eosin (H&E) staining (27). The authors concluded that the sensitivity and specificity of the technique was 97.5 and 98.5%, respectively. Similarly, Kalkanis et al. utilized a training set and subsequent validation series in order to employ Raman spectroscopy in distinguishing GBM from gray matter and necrosis. The authors reported 99.6 and 97.8% accuracy in the training and validation cohorts, respectively (28). An *in-vitro* study by Aguiar et al. revealed a sensitivity of 97.4% and a specificity of 100% in the diagnosis of GBM, medulloblastoma, and meningioma (29).

**Figure 2:**
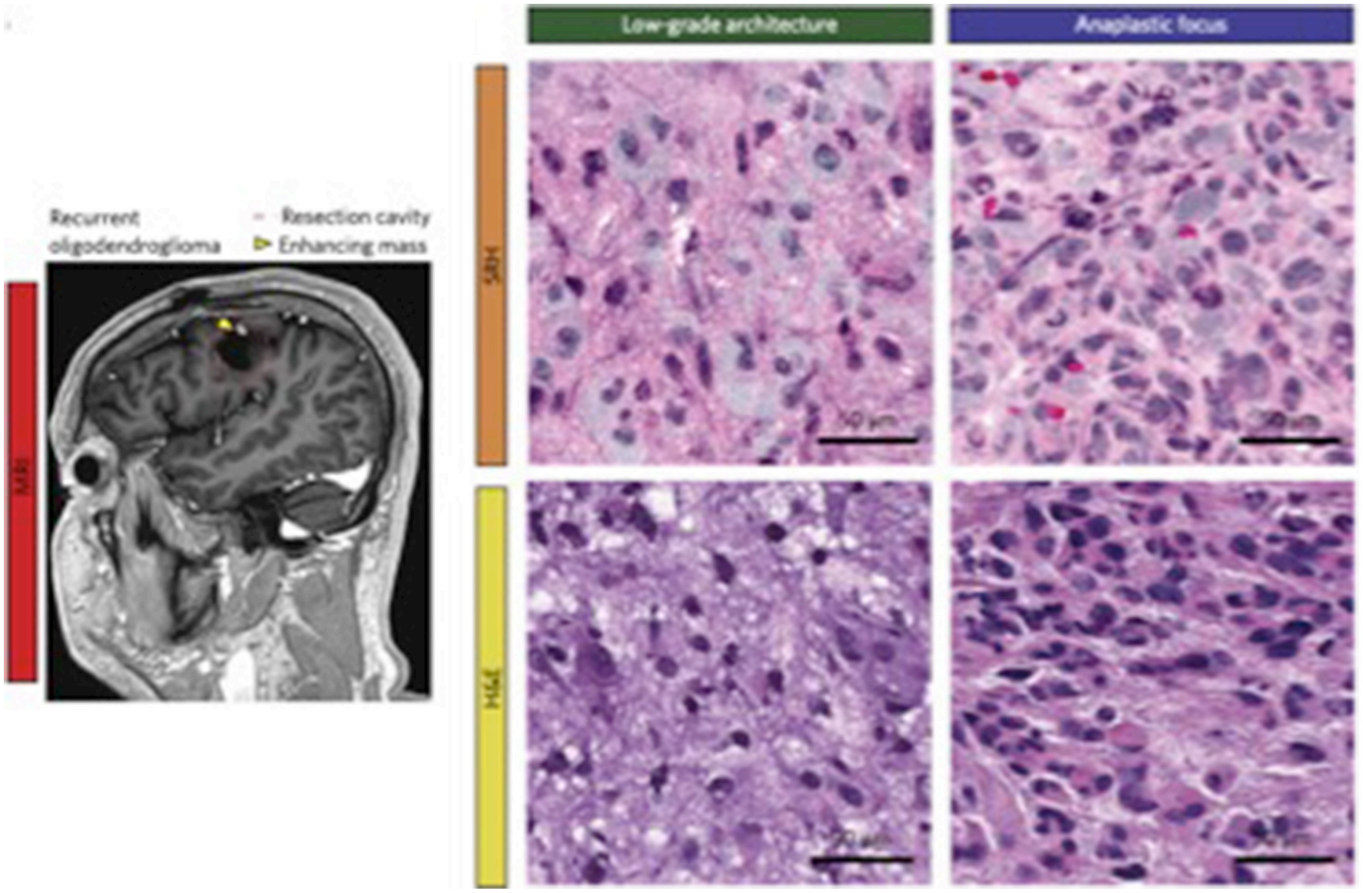
Depiction of Raman-stimulated histology slides alongside traditional haematoxylin and eosin for a patient with a history of recurrent oligodendroglioma [With permission from Orringer et al. (31)].

Other studies have revolved around the identification of important biomarkers that could be employed in distinguishing various tumor types. For instance, Zhou et al. demonstrated the utility of Raman spectroscopy in differentiating malignant tissue in 87 samples, revealing the presence of peaks corresponding to lactic acid and ATP in certain tumor tissues when compared to controls (30). These initiatives have been harnessed by investigations focused on their clinical application. Orringer et al. leveraged portable Raman spectroscopy to not only create virtual H&E-stained slides, but to create an algorithm that predicts brain tumor subtypes with 90% accuracy (31). A side-by-side comparison of images generated via Raman-stimulated histology and traditional haematoxylin and eosin staining is depicted in Figure 2. These techniques have already been replicated in the pediatric population, where Hollon et al. created a Raman spectroscopic-based algorithm that had near-perfect histologic concordance and could differentiate low and high-grade tumors with 100% accuracy (32). These findings demonstrate the substantial potential of algorithm-driven, handheld spectroscopy tools in providing valuable real-time data that could alter clinical management.

#### Clinical Studies

Comparatively few studies have examined the feasibility and utility of handheld Raman spectroscopy using real-time, *in-vivo* experimental design. Jermyn et al. employed a handheld probe in 17 patients with grade 2-4 gliomas and compared imaging findings with obtained biopsy specimens (33). The authors concluded that intraoperative Raman imaging facilitated the detection of cancer cells with an accuracy of 92%, compared to 73% using bright microscopy and MRI. The sensitivity and specificity in the differentiation of neoplasia from normal brain parenchyma were 93 and 91%, respectively. Desroches et al. developed a handheld Raman spectroscopy system and demonstrated its preliminary use in a swine brain biopsy model, followed by a human validation study involving 19 grade 2-4 glioma patients. The authors concluded that the handheld spectroscopy was able to detect malignancy during surgery with a sensitivity and specificity of 80 and 90%, respectively (34). Characteristics of selected studies on the role of Raman in diagnosing different types of brain tumors are presented in Table 1.

**Table 1:** Characteristics of selected studies reporting on the utility of Raman spectroscopy in tumor.

**References**	**Journal**	**Number of samples**	**Tumor histology**	**Relevant outcome metric**	**Rate**	**Notes**
**CLINICAL AND BASIC SCIENCE STUDIES**						
Ji et al. (27)	Sci Transl Med	22	Gliomas (both LGG and HGG)	Tumor infiltration (compared to H&E)	Sensitivity = 97.5% Specificity = 98.5%	–
Kalkanis et al. (28)	J Neurooncol	40	GBM	Differentiation of GBM from gray matter and necrosis	99.6 and 97.8% accuracy in training and validation cohorts, respectively	Utilized a training set with subsequent validation series
Aguiar et al. (29)	Photomed Laser Surg	172	GBM, medulloblastoma, meningioma	Diagnosis of tumor types	Sensitivity = 97.4% Specificity = 100%	–
Orringer et al. (31)	Nat Biomed Eng	30	Gliomas (both LGG and HGG) + meningioma, lymphoma, medulloblastoma, and metastases	Prediction of brain tumor subtypes (compared to H&E)	>92% accuracy	–
Hollon et al. (32)	Cancer Res	25	All tumor types	Prediction of brain tumor subtypes (compared to H&E)	92–96% accuracy	Pediatric brain tumor patients
Jermyn et al. (33)	Sci Transl Med	161	Gliomas (WHO grades II-IV)	Detection of malignancy (vs. bright microscopy and MRI)	Sensitivity = 93% Specificity = 91%	*In-vivo* experimental design
Desroches et al. (34)	Sci Rep	280	Gliomas (WHO grades II-IV)	Detection of malignancy (compared to H&E)	Sensitivity = 80% Specificity = 90%	Authors used handheld probe in swine brain biopsy model first, followed by human validation study

*GBM, Glioblastoma multiforme; HGG, High grade glioma; LGG, Low grade glioma; MRI, Magnetic resonance imaging; WHO, World Health Organization*.

#### Future Multimodal Techniques

Much of the recent literature on Raman spectroscopy in neurosurgical oncology revolves around the coupling of this technique to other, novel modalities in order to facilitate visualization. For instance, Neuschmelting et al. examined a combined approach of surface-enhanced Raman scattering and multispectral optoacoustic tomography in the detection of GBM cells in mouse brains. The authors reported that this new model exhibited a highly sensitive surface detection of infiltrating GBMs and could be transferrable to other animal models and potentially human trials (35). Meanwhile, Karabeber et al. demonstrated that the use of surface-enhanced Raman nanoparticles in the guidance of mouse GBM resection resulted in greater removal of residual tumor when compared to the use of white light alone (36). Jermyn et al. employed a combined, multimodal spectroscopic technique that incorporated fluorescence spectroscopy, diffuse reflectance spectroscopy, and Raman spectroscopy. The authors described high rates of accuracy, sensitivity, and specificity in differentiating brain, lung, colon, and skin malignancies *in situ* (37).

### Visualization in Fluorescence-Guided Surgeries

#### Basic Science

Numerous laboratory investigations have examined the feasibility and efficacy of handheld probes in detecting protoporphyrin IX. Kim et al. used a handheld, fiberoptic probe in order to quantify the fluorescence signal in *ex vivo* mouse brain tumors (38). They subsequently validated their technique using an *in vivo* rabbit brain tumor surgery model and were able to accurately differentiate tumor tissue from normal brain parenchyma. Cornelius et al. employed this same tool in order to examine its ability to differentiate different tumor types in humans (39). The authors concluded that the probe was a very sensitive tool in detecting 5-ALA-based fluorescence among 17 tumor biopsies, including differentiating between GBM and meningioma samples with the same precision as a laboratory spectrometer. Kairdolf et al. employed a low-cost hand-held spectroscopic instrument in order to compare the sensitivity of the handheld device with traditional surgical microscopes (25). They found that the handheld tool was, at a minimum, 3 times more sensitive and demonstrated strong specificity in differentiating tumor cells from normal brain tissue.

#### Clinical Studies

**Figure 3:**
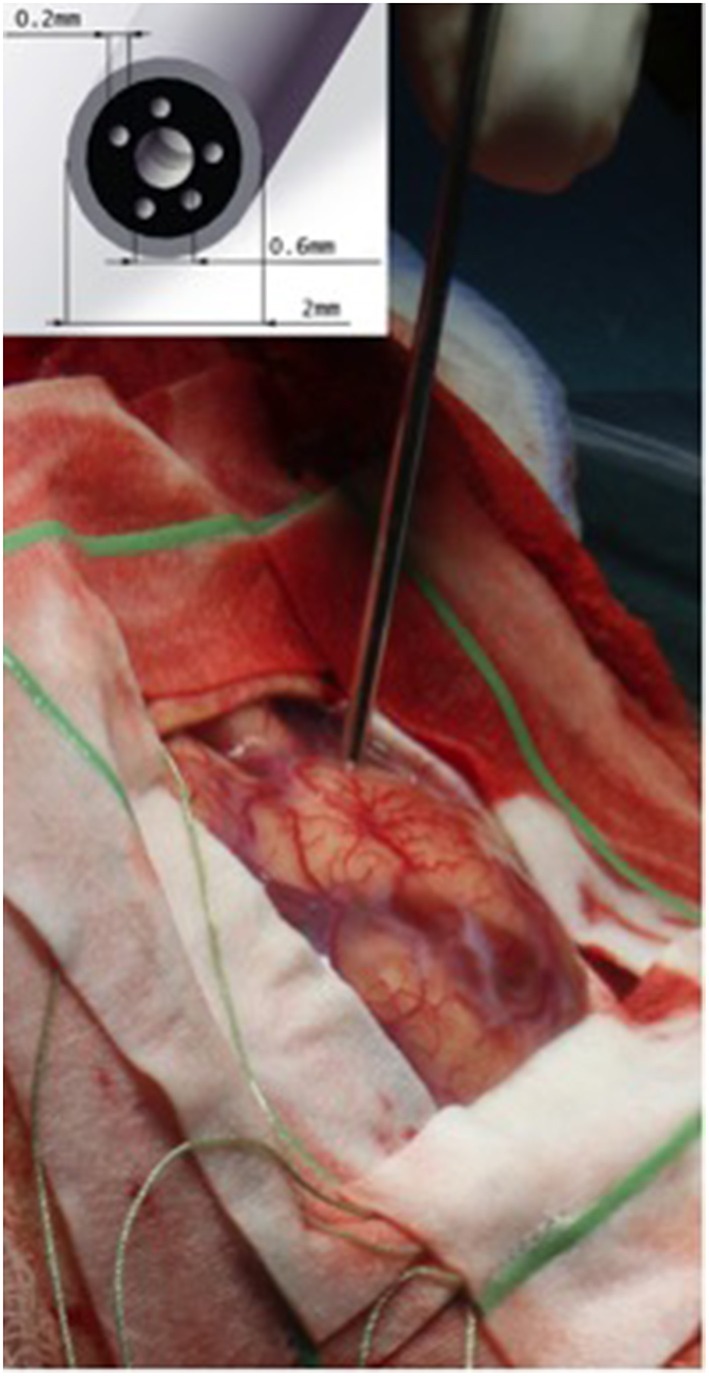
Intraoperative placement of the hand-held fluorescent probe on the cortical surface [With permission from Richter et al. (40)].

These handheld visualization devices have also been studied to assess their use and efficacy in the operating room. Haj-Hosseini et al. employed a handheld spectroscopic tool in order to make intraoperative measurements of white, gray, and known tumor tissue from patients undergoing GBM resection under 5-ALA fluorescence (26). The authors demonstrated the feasibility of this probe to accurately detect protoporphyrin IX fluorescence at the tumor margins *in vivo*. Richter et al. performed a study to directly compare the sensitivity of a hand-held fluorescence probe with fluorescence-guided microscopy in 16 patients undergoing resection of HGGs using 5-ALA. The authors highlighted that they were able to not only successfully integrate the tool into the operative routine, but that the handheld probe was superior to the microscope in sensitivity and detected additional tumor foci after the initial debulking using fluorescence-guided microscopy (40). The intraoperative use of the hand-held fluorescence probe is highlighted in Figure 3. These techniques have also been studied in other tumor types. Valdes et al. used a handheld probe tip in the intraoperative resection of low-grade gliomas, meningiomas, metastatic tumors, and GBM in order to compare quantitative fluorescence spectra across tumor types (41). They concluded that there was a significant difference in the quantitative measurements of protoporphyrin IX concentrations for all tumor groups when compared to normal brain tissue, as well as high sensitivity when compared to conventional fluorescence measurements.

## Discussion

### Tumor Identification Using Raman Probes

Overall, the use of handheld Raman spectroscopic imaging as an adjunct to neurosurgical resection is a promising development in brain tumor treatment and has been corroborated in a variety of clinical and basic science literature. The unique spectra allow for the differentiation of various tissue types. Numerous investigations have identified key biomarkers that can be employed to not only discriminate between tumor and normal parenchyma, but between various tumor types for both intra- and extra-axial lesions. Zhou et al., for instance, reported a detailed analysis of Raman spectra for an array of tumors, including GBM, acoustic neuroma, pituitary adenoma, and meningioma (42). This is turn has been used in several studies to create predictive algorithms with the capability to correctly predict histologic subtype with >90% accuracy (31, 32). The strong predictive capacity of this modality makes it useful in both the diagnosis and subsequent operative treatment of brain tumors.

### Intraoperative Advantages

Although numerous other modalities facilitate improved preoperative visualization of tumor, handheld spectroscopic tools offer the advantage of not only strong discriminatory capacity based on molecular footprint, but the ability to make physical adjustments intraoperatively. Unlike other visualization techniques, including intraoperative MRI, handheld probes can be angled away in order to inspect portions of the resection cavity that may not otherwise be visible. This may facilitate the identification of Raman-positive foci and removal of additional tumor. Karabeber et al. performed a study involving the surgical resection of GBM in mice, comparing resection with light microscopy, Raman microscopy, and hand-held Raman scanning (36). The authors demonstrated that the hand-held device was associated with a greater extent of resection, and noted several instances where the hand-held tool detected foci that were missed using microscopy, including the presence of tumor within the right lateral ventricle. This increased flexibility of hand-held devices offers the theoretical advantage of a potential combined therapy, where intraoperative Raman identification may be used alongside 5-ALA fluorescence-guided resection or conventional microscopic imaging. Although the application of 5-ALA has been shown to alter the Raman spectra of bladder tissues, this interference may be improved via fluorescence-subtraction algorithms, making a dual approach potentially viable *in-vivo* (43). Further studies are needed to explore the utility of combined modalities in the resection of brain tumors.

### 5-ALA Visualization

Other handheld tools have been developed in order to facilitate both visualization and quantification of protoporphyrin IX concentrations for patients undergoing tumor resection with 5-ALA. While the detection of fluorescence using traditional wide-field microscopy has been challenging for LGGs and other tumor types, handheld probes offer several advantages that can improve the utility of 5-ALA for these tumors. First, the ability to place these probes in close proximity to the tissue helps ameliorate the light scatter and suboptimal angle between traditionally employed microscopes and the resection bed (9). Second, the tools can be equipped with spectroscopic instrumentation that allows for the quantification of metabolite concentration, rather than relying on the subjective intraoperative assessment of visualized fluorescent signal (23). These advantages have been demonstrated in numerous studies highlighting the feasibility and efficacy of handheld probes *in vivo*. The ability to directly quantify fluorescence measurements in LGGs, meningiomas, and metastatic lesions, as well as the ability to intraoperatively distinguish them from brain parenchyma may increase the role of 5-ALA for these tumor types in the near future (41).

### Clinical Relevance

There is substantial literature reporting the relationship between the extent of resection and subsequent postoperative outcomes in patients with brain tumors (1–3). Identification of microscopic disease and residual tumor remains the primary challenge in neuro-oncology. As such, all tools that facilitate the intraoperative visualization of lesions have the potential to provide clinical benefit to patients. Despite this, however, there is a dearth of literature examining the relationship between the use of spectroscopic handheld tools and improved postoperative clinical endpoints. Additional studies are needed to examine the association between intraoperative Raman spectroscopy, protoporphyrin IX quantification tools, extent of resection, and OS/PFS.

## Conclusions

There is significant evidence demonstrating the utility of Raman spectroscopic imaging in identifying areas of malignancy in both human and animal specimens. Several studies have highlighted the use of concomitant algorithms in accurately diagnosing histologic tumor subtype and the feasibility of using handheld spectroscopy tools in the operative setting. In addition, numerous studies have demonstrated the efficacy of handheld probes in the operative quantification of protoporphyrin IX levels for patients undergoing 5-ALA fluorescence-guided resection. Further studies exploring the relationship between *in-vivo* spectroscopic use, extent of resection, and postoperative survival are needed to better assess the impact of these tools on patient outcomes.

## Author Contributions

All authors listed have made a substantial, direct and intellectual contribution to the work, and approved it for publication.

### Conflict of Interest Statement

CH is a consultant for NXDC and Synaptive Medical Inc. He will receive royalties from NXDC. He has also received speaker fees by Carl Zeiss and Leica. The remaining author declares that the research was conducted in the absence of any commercial or financial relationships that could be construed as a potential conflict of interest.
